# The Chemerin/CMKLR1 Axis Is Involved in the Recruitment of Microglia to Aβ Deposition through p38 MAPK Pathway

**DOI:** 10.3390/ijms23169041

**Published:** 2022-08-12

**Authors:** Yanqing Chen, Zhen Liu, Ping Gong, Haibo Zhang, Yijun Chen, Songquan Yao, Wei Li, Yan Zhang, Yang Yu

**Affiliations:** School of Pharmacy, Shanghai Jiao Tong University, Shanghai 200240, China; ts_chen@foxmail.com (Y.C.); lz974639707@sjtu.edu.cn (Z.L.); gongpingping@ccmu.edu.cn (P.G.); zhb_zhanghaibo@sjtu.edu.cn (H.Z.); chenyj98@sjtu.edu.cn (Y.C.); yaosongquan@sjtu.edu.cn (S.Y.); liweishen@sjtu.edu.cn (W.L.); zhangyan_sjtu@sjtu.edu.cn (Y.Z.)

**Keywords:** Alzheimer’s disease, chemerin, CMKLR1, chemR23, microglia, migration, p38 MAPK

## Abstract

The accumulation of microglia around senile plaques is one of the pathological features of Alzheimer’s disease (AD). Chemerin is an adipokine with immune-modulating properties. Our previous study showed that chemokine-like receptor 1 (CMKLR1), the receptor for chemerin, is also a functional receptor of Aβ. However, it remains unclear whether and how the chemerin/CMKLR1 axis affects the migration of microglia. The impact of CMKLR1 on microglial activation and recruitment toward Aβ deposits was examined in APP/PS1 mice mated with CMKLR1 knockout (*CMKLR1^−/−^*) mice. CMKLR1 deficiency reduced the number of microglia around Aβ deposits in aged APP/PS1-*CMKLR1*^−/−^ mice compared with APP/PS1 mice. Chemerin expression was significantly decreased in the hippocampus and cortex of aged APP/PS1 mice compared with WT mice. In vitro assays demonstrated that activation of the chemerin/CMKLR1 axis promoted the migration of primary cultures of microglia and murine microglial N9 cells. Mechanistic studies found that chemerin/CMKLR1 induced polarization and protrusion formation of microglia by promoting the remodeling of actin filaments and microtubules, and Golgi apparatus reorientation. The inhibition of p38 MAPK attenuated the promotion of the chemerin/CMKLR1 axis on microglial migration and polarization. In addition, chemerin inhibited Aβ-induced microglial clustering. The inhibition of p38 MAPK alleviated the suppressive effect of chemerin on Aβ-induced microglial aggregation. Our data indicate that the chemerin/CMKLR1 axis is involved in the migration and recruitment of microglia to senile plaques via the p38 MAPK pathway. Modulation of the chemerin/CMKLR1 axis is a potential new strategy for AD therapy.

## 1. Introduction

Alzheimer’s disease (AD) is a chronic neurodegenerative disorder characterized by progressive memory loss and cognitive impairment [1]. Extracellular senile plaques (SPs) formed by aggregated amyloid β (Aβ) in the brain are one of the main pathological features of AD [2]. Aggregated Aβ plays a pivotal role in the progression of AD through neuroinflammatory effects [3,4]. The brain cells related to AD neuroinflammation mainly include microglia and astrocytes. Microglia are the primary immune cells in the central nervous system (CNS) and the main mediator of innate immune response to invading pathogens and neuronal injuries [5]. Microglia are activated in AD and have been found to surround Aβ plaques [3]. It is believed that the neuroinflammatory response induced by microglia is a double-edged sword in the pathological progression of AD. Activated microglia migrating and recruited to surround Aβ plaques are believed to clear Aβ at the early stages of AD, but as the disease progresses, the effectiveness of phagocytosis decreases. On the other hand, due to the sustained activation of accumulated Aβ or Aβ deposits, microglia maintain their ability to produce proinflammatory cytokines, chemokines, and free radicals, which contribute to further production and accumulation of Aβ [6]. Therefore, mediation on the migration and activation of microglia may be one of the key strategies for preventing neurodegenerative diseases.

Chemerin is an adipokine. It is synthesized and secreted by several tissues and cells, including adipose tissue [7], liver [8,9], spleen [9], lymph nodes [10], and immune cells [11,12,13]. Previous studies have shown that chemerin is associated with obesity, insulin resistance, metabolic diseases, and inflammation [14,15]. Three natural receptors for chemerin have been identified so far: chemokine-like receptor 1 (CMKLR1 or chemR23), chemokine (CC-motif) receptor-like 2 (CCRL2), and G protein-coupled receptor 1 (GPR1) [16,17,18]. However, only CMKLR1 has been reported to be responsible for the inflammation and chemotaxis of immune cells induced by chemerin [16,19]. CMKLR1 is a seven transmembrane-domain GPCR (G protein-coupled receptor) that has been found in hematopoietic tissues; adipocytes; endothelial cells; and specific immune cells such as immature dendritic cells (DCs), macrophages, and cytotoxic natural killer (NK) cells [20]. Recently, accumulating evidence has indicated that the chemerin/CMKLR1 axis may play multiple roles in regulating inflammation, metabolism, and cancerogenesis [14,15,20]. Chemerin and CMKLR1 are also expressed in the brains of mice and humans [20,21]. However, little is known about their role in neurodegenerative diseases. A study found that human recombinant chemerin (rh-chemerin) attenuates germinal matrix hemorrhage (GMH)-induced neuroinflammation by promoting the CMKLR1/CAMKK2/AMPK pathway [22]. Our previous study found that CMKLR1 expression increases in the brains of AD patients and AD mice [21]. We also found that Aβ binds specifically to CMKLR1 in stably transfected rat basophilic leukemia cells (CMKLR1-RBL cells). Aβ treatment induces the migration of CMKLR1-RBL cells and primary cultures of mouse microglial cells [21]. These results indicate that CMKLR1 is also a functional receptor for Aβ. Our recent research also found that CMKLR1 deficiency reduces mortality, improves the cognitive deficits of AD mice, and attenuates tau hyperphosphorylation in the brains of AD mice in vivo and in neuronal cells in vitro. Another mechanism study discovered that the expression of CMKLR1 on neurons affects tau phosphorylation by participating in tau spreading [23]. However, we have not explored whether or how CMKLR1 participates in the pathological process of AD by regulating neuroinflammation. In addition, there is no report on the function of the chemerin/CMKLR1 axis in AD. Previous studies have found that p38 MAPK activity increases in the brains of patients with early AD. Increased phosphorylated p38 is related to neuritic plaques, neurofilaments, and neurofibrillary tangle neurons [24]. Evidence suggests that p38 MAPK may be involved in neuroinflammatory responses in AD by mediating Aβ-induced IL-6 production [25]. Interestingly, site-specific phosphorylation of tau alleviates Aβ-induced postsynaptic excitotoxicity via neuronal p38 MAPK [26]. Our recent study found that p38 MAPK is activated and involved in regulating the inhibition of serum amyloid A on astrocyte migration [27]. All of these findings indicate that p38 MAPK may play a pivotal role in the pathogenesis of AD.

In this study, we hypothesize that the chemerin/CMKLR1 axis may play a role in the migration of microglia, thereby regulating their recruitment to Aβ plaques. We first established APP/PS1 transgenic and CMKLR1 knockout (APP/PS1-*CMKLR1*^−/−^) mice to verify how CMKLR1 affects the migration and recruitment of microglial cells to Aβ deposits. We found that CMKLR1 deficiency reduced the colocalization of activated microglia with Aβ plaques in the hippocampus and cortex of APP/PS1-*CMKLR1*^−/−^ mice compared with the APP/PS1 mice, indicating that the presence of CMKLR1 promotes the migration of microglial cells to Aβ plaques. Interestingly, the expression of chemerin was significantly decreased in the brain of APP/PS1 mice compared with in that of WT mice. In vitro studies verified that activation of the chemerin/CMKLR1 axis promoted the migration and polarization of microglia by regulating actin and microtubule remodeling, and Golgi reorientation by activating the p38 MAPK pathway. In addition, chemerin inhibited Aβ-induced microglial aggregation. The inhibition of p38 MAPK alleviated the blocking effect of chemerin on Aβ-induced microglial accumulation.

## 2. Results

### 2.1. CMKLR1 Deficiency Decreases Microglia Colocalization with Aβ Plaques in the Brain of APP/PS1 Transgenic Mice

**Figure 1:**
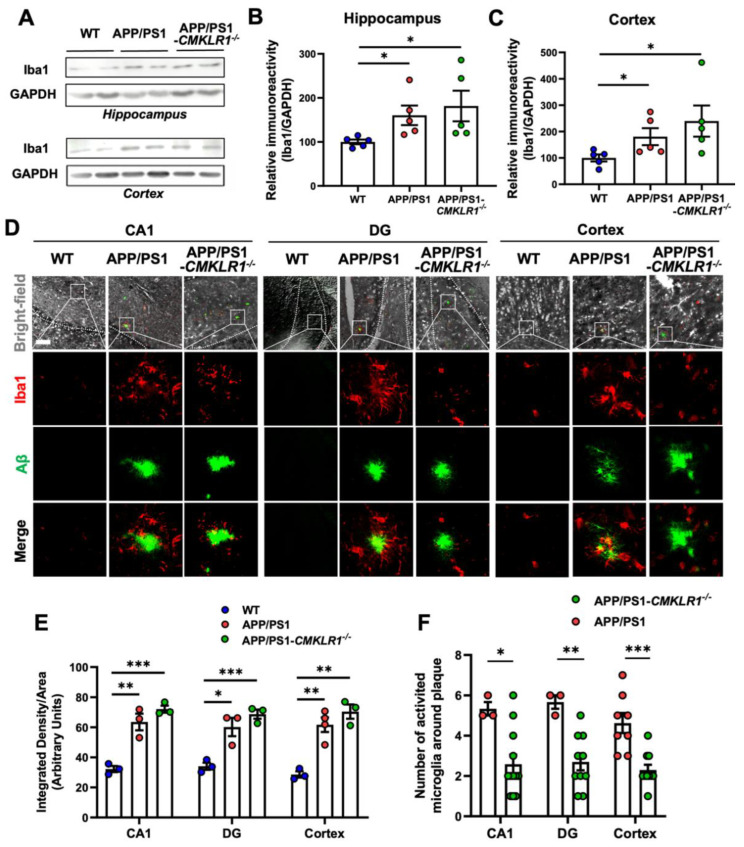
CMKLR1 deficiency reduces the number of activated microglia around Aβ plaques in brain of APP/PS1 transgenic mice. (**A**) Representative Western blot showing the expression of Iba1 in the hippocampus and cortex in 9-month-old WT, APP/PS1, and APP/PS1-*CMKLR1*^−/−^ mice. (**B**,**C**) Quantification of immunoreactivity of the blots, normalized against GAPDH. (**D**) Serial sections of 9-month-old WT, APP/PS1, and APP/PS1-*CMKLR1*^−/−^ mice brains were stained for Aβ deposits (green fluorescence) and microglia (red fluorescence) as described in the “Materials and methods” section. The scale bar in the upper left panel is 100 μm. Selected areas are enlarged by 25 times and shown as combined as well as individual fluorescence stains. Quantification of the fluorescence of microglia (**E**), and the number of microglia (**F**) around Aβ deposits was shown. The results are expressed as the mean ± SEM on each region by using at least 3 mice in each group. * *p* < 0.05, ** *p* < 0.01, *** *p* < 0.001.

We have shown that CMKLR1 is a functional receptor of Aβ [21]. CMKLR1 deficiency increases Aβ deposits in AD mice, reduces mortality, and improves the cognitive impairment of AD mice [23]. Since neuroinflammation plays a pivotal role in AD pathogenesis and microglia are important immune cells in CNS, we validated the effect of CMKLR1 on the migration and activation of microglia in the AD mouse brain. We generated CMKLR1-deficient APP/PS1 transgenic mice (APP/PS1-*CMKLR1*^−/−^) by crossing APP/PS1 double transgenic mice (on a C57BL/6J background) with *CMKLR1*^−/−^ mice (also on a C57BL/6J background). We analyzed the brains of APP/PS1-*CMKLR1*^−/−^ mice at the age of 9 months in comparison with APP/PS1 mice of the same age. To examine the effect of CMKLR1 deficiency on the activation of microglia, the level of Iba1 was detected by Western blot. As shown in Figure 1A–C, Iba1 expression was significantly increased in the hippocampus and cortex of APP/PS1 mice versus WT mice. CMKLR1 deficiency resulted in a trend of increasing expression of Iba1 in APP/PS1-*CMKLR1*^−/−^ mice compared with APP/PS1 mice, but there was no statistically significant difference. Additionally, immunofluorescence was also conducted to test the effect of CMKLR1 deletion on the activation of microglia. Consistent with the results of the Western blot, microglia were significantly activated in the hippocampus (CA1 and DG regions) and cortex of the APP/PS1 mice, whereas CMKLR1 deletion did not affect the activation of microglia compared with that in the APP/PS1 group (Figure 1D,E).

Next, we explored whether CMKLR1 is involved in the migration of microglia towards Aβ deposition. Our previous study found that CMKLR1 expression was significantly enhanced in AD mouse brain by immunofluorescence staining [23]. In this study, we also observed a significant increase in CMKLR1 expression in the hippocampus and cortex of APP/PS1 mice by using Western blot (Supplementary Figure S1A,B), consistent with our previous results. Then, the frozen slices of brain tissue were double-stained for immunofluorescence with anti-ionized calcium-binding adapter molecule 1 (anti-Iba1, red fluorescence) with thioflavin-S (anti-Aβ plaque, green fluorescence) to investigate the colocalization of microglia with Aβ deposits in the mouse brain. Immunofluorescence staining and quantitative analysis revealed a significant decrease in the number of activated microglia colocalized with Aβ deposits in the hippocampus (CA1 and DG regions) and cortex of APP/PS1-*CMKLR1*^−/−^ mice compared with that of the APP/PS1 mice (Figure 1D,F). These results suggest that the deficiency of CMKLR1 inhibits the migration of microglia to Aβ plaques.

**Figure 2:**
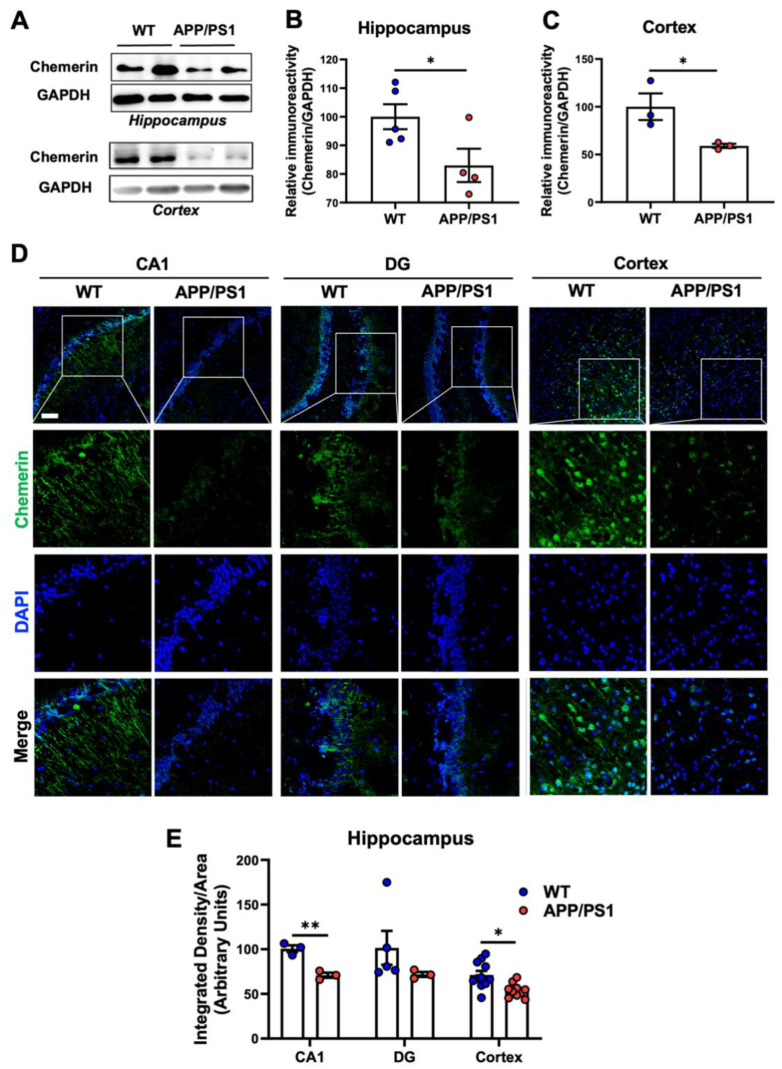
Decrease of chemerin in the hippocampus and cortex of APP/PS1 transgenic mice. (**A**) Representative Western blot showing the expression of chemerin in the hippocampus and cortex in 9-month-age WT and APP/PS1 mice. (**B**,**C**) Quantification of immunoreactivity of the blots, normalized against GAPDH. (**D**) Serial sections of 9-month-old WT and APP/PS1 transgenic mouse brains were stained for chemerin using a rabbit anti-chemerin polyclonal antibody and then further treated with Alexa Fluor 488-conjugated anti-rabbit IgG (green fluorescence). Cell nuclei were stained with DAPI (blue fluorescence). Immunofluorescence was examined under a confocal laser-scanning microscope. Scale bar, 50 μm. Selected areas are enlarged by 4 times and shown as combined as well as individual fluorescence stains. (**E**) Quantification of the chemerin fluorescence was shown. The results are expressed as the mean ± SEM on each region by using at least 3 mice in each group. * *p* < 0.05, ** *p* < 0.01.

Furthermore, we detected changes in chemerin expression in the brains of AD mice. A significant decrease in chemerin expression was observed in the hippocampus and the cortex of APP/PS1 mice aged 9 months compared with WT mice of the same age by Western blot (Figure 2A–C) and immunofluorescence staining (Figure 2D,E). These data suggest that the chemerin/CMKLR1 axis participates in the pathological process of AD by regulating the migration of microglia.

### 2.2. Chemerin/CMKLR1 Axis Induces the Migration of Microglia

**Figure 3:**
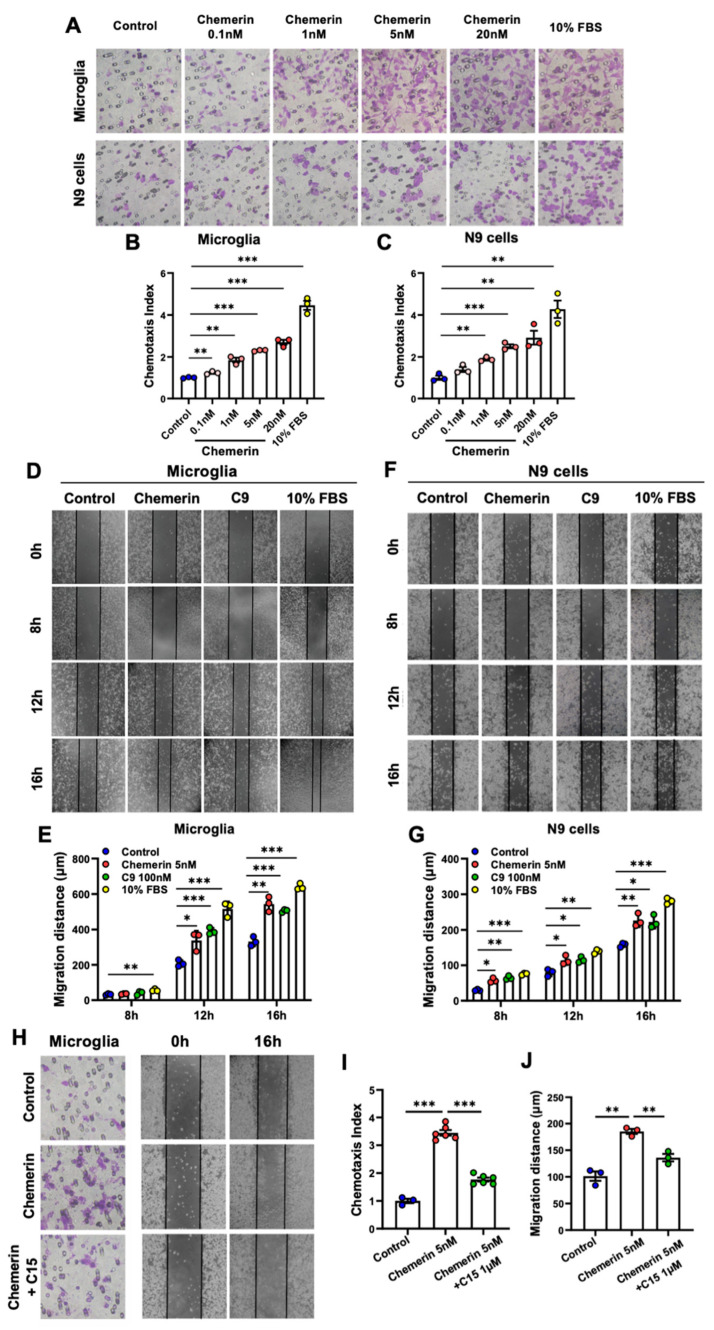
Chemerin/CMKLR1 axis promotes the migration of microglia in Boyden chamber migration and scratch-wound assay. (**A**) Primary cultures of microglia and murine microglial N9 cells were incubated for 12 h with chemerin (0.1–20 nM) or 10% FBS, and the migration of the cells was evaluated by 48-well chemotaxis chambers. Representative images of migrated cells on membrane filters are shown. (**B**,**C**) The Quantified data are shown. Magnification, ×400. Primary microglia and N9 cells were treated with chemerin (5 nM), C9 (100 nM), or 10% FBS; then, the migration of the cells was detected by scratch-wound assay. The microglia were photographed at 0 h, 8 h, 12 h, and 16 h. Representative images of migrated primary microglia and N9 cells are shown in (**D**,**F**), and quantified data are shown in (**E**,**G**), respectively. Magnification, ×100. (**H**) Primary microglia were incubated with chemerin (5 nM) with or without a 15 min pretreatment with C15 (1 μM). After 16 h incubation, the migration of microglia was detected by 48-well chemotaxis chambers and scratch-wound assay. Magnification, ×400 and ×100, respectively. The quantified data were shown in (**I**,**J**). The results are expressed as the mean ± SEM from three separate experiments, each in at least triplicate. * *p* < 0.05, ** *p* < 0.01, *** *p* < 0.001.

The Boyden chamber assay was conducted using primary cultures of rat microglia and mouse microglial N9 cells to verify the role of chemerin/CMKLR1 in microglial migration. Chemerin (0.1–20 nM) was added in the lower chamber well as the chemoattractant, and the cells were seeded to the upper wells. After 12 h, the cells that migrated through the filter were counted. The results showed that chemerin significantly induced the migration of primary cultures of microglia and N9 cells in a dose-dependent manner (Figure 3A–C). C9 is a nonapeptide derived from chemerin that retains most of the agonistic activity of chemerin. C9 and 10% FBS (as a positive control) were also found to induce the migration of these microglial cells (Supplementary Figure S2A–C). Additionally, the results of the MTT assay showed that chemerin (20 nM) or C9 (100 nM) treatment alone for 16 h did not affect the viability of primary cultures of microglia and N9 cells (Supplementary Figure S3A,B).

In addition to the Boyden chamber assay, the scratch-wound assay was also performed to verify the effect of chemerin on the migration of microglia. Primary cultures of microglia and N9 cells were scratched and treated with 5 nM chemerin, 100 nM C9, or 10% FBS for 8 h, 12 h, or 16 h. Images were taken at 0 h, 8 h, 12 h, and 16 h, and migration distance was quantified. Significant promotion of wound closure was observed in these microglial cells treated with chemerin, C9, or 10% FBS compared with cells treated with DMEM alone (Figure 3D–G), consistent with the results of the Boyden chamber assay. All of these results suggest that chemerin promotes the migration of microglia.

Next, to examine whether chemerin induces the migration of microglial cells via activating CMKLR1, the C15 peptide was used. C15 is a chemerin-derived 15-residue peptide that lacks the agonistic activity of chemerin [17]. As shown in Figure 3H–J, the Boyden chamber and scratch-wound assay showed that pre-treatment with C15 inhibited the migration of primary cultures of microglia induced by chemerin. These results indicate that the chemerin/CMKLR1 axis promotes the migration of microglia.

### 2.3. Chemerin/CMKLR1 Promotes Actin and Microtubule Remodeling, and Golgi Reorientation in Microglia

**Figure 4:**
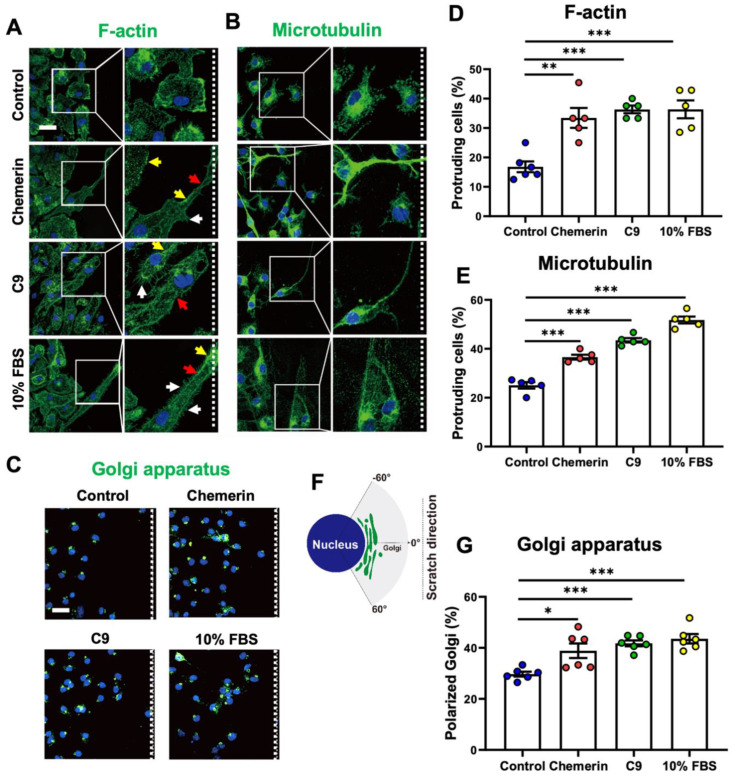
Chemerin promotes remodeling of actin filaments and microtubules, and reorientation of Golgi apparatus in microglia with scratch-wound assay. (**A**,**B**) Primary cultures of microglia were incubated with chemerin (5 nM), C9 (100 nM), or 10% FBS for scratch-wound assay. At 16 h after scratching, microglia were stained with anti-FITC-phalloidin (F-actin, green fluorescence) or anti-α-tubulin (Microtubule) polyclonal antibody and further treated with Alexa Fluor 488-conjugated anti-rabbit IgG (green fluorescence). The morphological changes in microglia could be identified: long protrusions (red arrow), lamellipodia-like structures (yellow arrow), and filopodia-like structures (white arrow). (**E**) At 12 h after scratching, the cells were stained with anti-GM130 (Golgi apparatus) polyclonal antibody and then further treated with Alexa Fluor 488-conjugated anti-rabbit IgG (green fluorescence). The cell nuclei were stained with DAPI (blue fluorescence). The scale bar in the upper left panel is 50 μm. The white dashed lines indicate the direction of the wound. The selected areas were enlarged by four times. (**C**,**D**) For F-actin and microtubule staining, primary microglia were scored as protruding when their protrusions were at least 4 times longer than wide. (**F**,**G**) For Golgi apparatus staining, the percentage of primary microglia with the Golgi apparatus in the forward-facing 120° sectors toward the wound center was measured in wound-edge cells. The results are expressed as the mean ± SEM from three separate experiments, each in at least triplicate. * *p* < 0.05, ** *p* < 0.01, *** *p* < 0.001.

Cell polarization is a necessary step in cellular movement and directed migration [27]. Then, we further examined the effect of chemerin/CMKLR1 on microglial polarization using the scratch-wound assay. Primary microglia were scratched and treated with 5 nM chemerin, 100 nM C9, or 10% FBS for 16 h. The cells were stained with anti-phalloidin for F-actin or anti-α-tubulin for the microtubules. The results showed that chemerin or C9 treatment induced morphological changes in the microglial membrane such as long protrusions (red arrow) towards the wound center with increased fan-shaped actin-rich lamellipodia-like structures (yellow arrow) and filopodia-like structures (white arrow) (Figure 4A,B). These morphological changes have been reported to be the main features of microglial migration [28]. However, in the control group, the microglia remained small cell bodies with short protrusions (Figure 4A,B). Microglia were scored as protruding cells when their protrusions were at least four times longer than wide. A quantitative analysis of F-actin and microtubule staining revealed a significant increase in the number of protruding cells at the wound edge in primary cultures of microglia treated with chemerin or C9 compared with the control group (Figure 4C,D). These results suggest that chemerin/CMKLR1 promotes the polarization of microglia by inducing the remodeling of actin and microtubule networks in the protrusions.

Golgi apparatus reorientation is another requirement in cell migration. We further explored the regulation of chemerin on the Golgi apparatus in the scratch-wound assay. Primary cultures of microglia were stained with anti-GM130 for the Golgi apparatus. The reorientation of Golgi in wound-edge cells was detected and analyzed at 12 h after scratching. As shown in Figure 4E, the white dashed lines indicate the direction of the wound. Primary microglia with the Golgi apparatus towards the wound center (in the forward-facing 120° sectors, Figure 4F) was measured. In the control group, about 30% of the wound-edge microglial cells have Golgi (green fluorescence) towards the direction of the wound center (Figure 4E–G). Chemerin or C9 treatment increased the number of microglia with the Golgi apparatus towards the wound center compared with the control group. All of these data indicate that chemerin/CMKLR1 promotes microglia migration and polarization by inducing actin and microtubulin remodeling, and Golgi reorientation.

### 2.4. p38 MAPK Pathway Is Involved in the Promotion of Chemerin/CMKLR1 on the Migration and Polarization of Microglia

**Figure 5:**
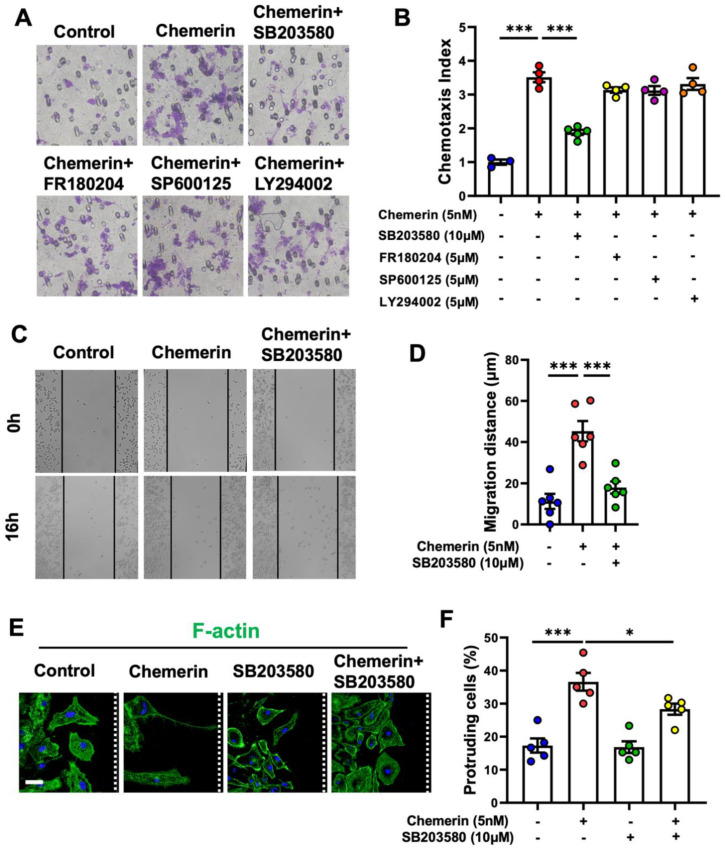
Inhibiting p38 suppresses the promotion of chemerin on the migration and polarization of primary microglia. (**A**) Primary cultures of microglia were incubated with chemerin (5 nM) with or without 15 min pretreatments with SB203580 (10 μM), FR180204 (5 μM), SP600125 (5 μM), and LY294002 (5 μM). After 12 h of incubation, the migration of the cells was detected by 48-well chemotaxis chambers. Magnification, × 400. The quantified data are shown in (**B**). Microglial N9 cells were treated with chemerin (5 nM) with or without a 15 min pretreatment with SB203580 (10 μM); then, the migration of microglia was detected by scratch-wound assay. The cells were photographed at 0 h and 16 h. Representative images of migrated cells are shown in (**C**), and quantified data are shown in (**D**). (**E**) Primary microglia were incubated with chemerin (5 nM) with or without a 15 min pretreatment with SB203580 (10 μM) for scratch-wound assay. At 16 h after scratching, the cells were stained with anti-FITC-phalloidin (F-actin, green fluorescence). Cell nuclei were stained with DAPI (blue fluorescence). Scale bar, 50 μm. The white dashed lines indicate the direction of the wound. (**F**) For F-actin staining, primary microglia were scored as protruding when their protrusions were at least 4 times longer than wide. The results are expressed as the mean ± SEM from three separate experiments, each in at least triplicate. * *p* < 0.05, *** *p* < 0.001.

Studies have found that activated mitogen-activated protein kinases (MAPKs) and phosphatidylinositol 3-kinases (PI3K) are involved in regulating cell polarization and migration [29,30]. Kaur et al. reported that chemerin dose-dependently activates MAPKs (p38 and ERK1/2) and PI3K/Akt pathways in human endothelial cells (ECs) [31]. Our previous studies showed that Aβ42 induces CMKLR1-dependent cell migration by activating the ERK1/2 and Akt pathways [21]. Here, we used selective inhibitors of MAPKs and PI3K kinases to verify whether these pathways participate in the promotion of chemerin/CMKLR1 on the migration and polarization of primary cultures of microglia. We first excluded the effects of these inhibitors themselves on cell viability and migration. MTT and Boyden chamber assay found that SB203580 (a p38 inhibitor, 10 μM), FR180204 (an ERK1/2 inhibitor, 5 μM), SP600125 (a JNK inhibitor, 5 μM), or LY294002 (a PI3K inhibitor, 5 μM) treatment alone for 16 h did not affect the viability and migration of primary cultures of microglia (Supplementary Figures S4 and S5). The concentration of inhibitors was chosen as the above assays. Then, we pretreated cells with these inhibitors and observed their effect on chemerin-induced microglial migration. The results showed that 15 min pre-treatment with SB203580 (10 μM) significantly inhibited the promotion of chemerin on the migration of microglia in the Boyden chamber assay and scratch-wound assay (Figure 5A–D and Figure S6). However, FR180204 (5 μM), SP600125 (5 μM), or LY294002 (5 μM) pretreatment did not affect chemerin-induced microglial cell migration. These data indicate that p38 MAPK participates in the regulation of chemerin on the migration of microglia. In addition, we confirmed whether p38 MAPK was involved in chemerin/CMKLR1 inducing the polarization of microglia. The results showed that SB203580 pretreatment significantly inhibited chemerin-induced actin remodeling and decreased protruding cells at the wound edge in primary cultures of microglia (Figure 5E,F).

### 2.5. p38 MAPK Pathway Is Involved in the Suppressive Effects of Chemerin on Aβ-Induced the Aggregation of Microglia

**Figure 6:**
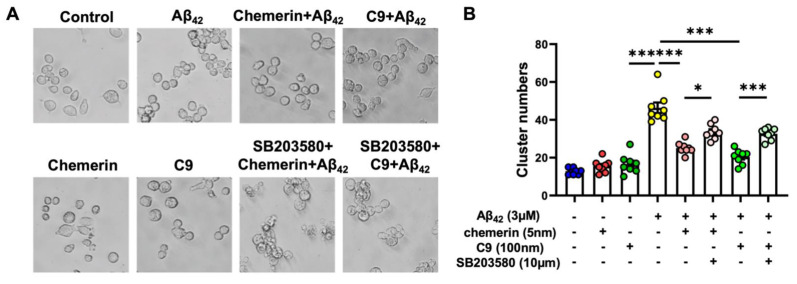
Activation of Chemerin/CMKLR1 axis attenuated Aβ-induced microglial aggregation. (**A**) Microglial N9 cells were incubated with chemerin (5 nM) or C9 (100 nM) for 30 min with or without a 15 min pretreatment with SB203580 (10 μM), followed by 3 μM aggregated Aβ_42_ for 24 h. Representative images of control, Aβ_42_, or Aβ_42_ with chemerin or C9 incubation with or without SB203580. Quantification of the number of microglial cells cluster is shown in (**B**). Images were taken from four random fields for each well. The results are expressed as the mean ± SEM from three separate experiments, each in duplicate or triplicate. * *p* < 0.05, *** *p* < 0.001.

Since chemerin expression was significantly decreased in the brain of AD mice and CMKLR1 deficiency reduced the migration of microglial cells to Aβ plaques. In vitro studies verified that activation of the chemerin/CMKLR1 axis promoted the migration and polarization of microglia by regulating actin and microtubule remodeling, and Golgi reorientation by activating the p38 MAPK pathway. We verified in vitro whether chemerin could mediate the migration of microglia to Aβ and whether the mediation was involved in the p38 MAPK signaling pathway. As shown in Figure 6A,B, Aβ_42_ induced significant aggregation of microglial N9 cells. Chemerin (5 nM) or C9 (100 nM) treatment significantly suppressed the accumulation of microglial N9 cells. Furthermore, the inhibition effect of chemerin or C9 on microglial aggregation induced by Aβ_42_ was attenuated by the p38 MAPK inhibitor (10 μM SB203580). These findings indicate that chemerin/CMKLR1 reduces Aβ-induced microglial aggregation by regulating the p38 MAPK pathway.

## 3. Discussion

**Figure 7:**
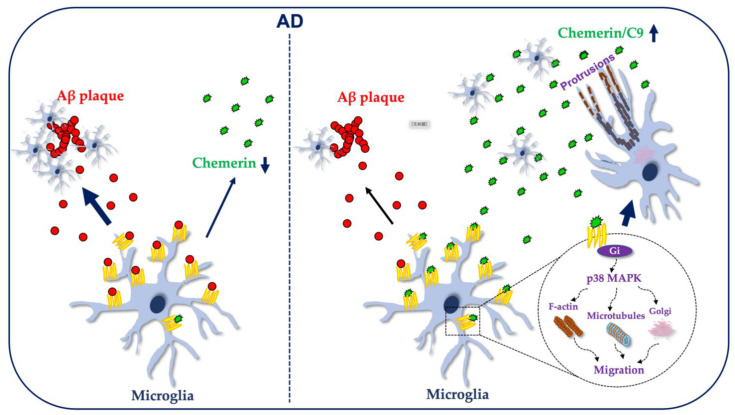
Schematic representation of the involvement of the chemerin/CMKLR1 axis in the recruitment of microglia to Aβ plaques. Under AD pathological conditions, the chemerin/CMKLR1 axis is weakened and the Aβ/CMKLR1 axis is overactivated, which leads to the migration and recruitment of microglial cells to Aβ plaques. Strengthening the chemerin/CMKLR1 axis promotes the migration and polarization of microglia by inducing actin filament and microtubule remodeling, and Golgi reorientation. p38 MAPK pathway activation serves to promote the chemerin/CMKLR1 axis on the migration and polarization of microglia.

Activated microglia have been observed to accumulate around Aβ plaques in postmortem human tissue from AD patients and in AD animal models [3]. The mechanism and causes of this pathological phenomenon are not yet fully understood. This study demonstrates for the first time that the chemerin/CMKLR1 axis is involved in the migration and recruitment of microglia to Aβ plaques in vivo. Mechanistic studies have shown that the chemerin/CMKLR1 axis promotes the migration and polarization of microglia by regulating actin and microtubule remodeling, and Golgi reorientation. Inhibition of the p38 MAPK abolishes the promotion of chemerin/CMKLR1 on microglial cell migration and polarization. In addition, chemerin inhibited Aβ-induced microglial aggregation. The inhibition of p38 alleviated the blocking effect of chemerin on Aβ-induced microglial aggregation. These findings provide new evidence for the role of CMKLR1 in AD and indicate that regulation of the chemerin/CMKLR1 axis may be a new strategy for the treatment of AD (Figure 7).

In this study, we used the APP/PS1 double transgenic mice, the most widely employed experimental model of AD, to verify the effect of chemerin/CMKLR1 on the migration and activation of microglial cells in vivo. We have found that CMKLR1 deficiency reduces the number of activated microglia colocalized with Aβ deposits in the hippocampus and cortex of APP/PS1-*CMKLR1^−/−^* mice compared with that in APP/PS1 mice, indicating that the presence of CMKLR1 promotes the migration and recruitment of microglia to Aβ plaques. However, interestingly, the expression of chemerin decreased in APP/PS1 mice compared with that in WT mice. Our previous and current studies have also found that CMKLR1 is upregulated in the brain of AD mice [21,23]. CMKLR1 is a functional receptor for Aβ, and Aβ/CMKLR1 can also induce the migration of microglia, although not producing calcium flux [21]. These results suggest that, under AD pathological conditions, the chemerin/CMKLR1 axis is weakened while the Aβ/CMKLR1 axis is overactivated, which leads to the migration and recruitment of microglia to Aβ plaques (Figure 7). In addition, we also found that CMKLR1 deficiency did not affect the activation of microglia. Our previous results have shown that Aβ/CMKLR1 does not seem to induce microglia to release inflammatory factors such as interleukin 6 (IL-6), IL-12, and inducible nitric oxide synthase (iNOS) [4]. Zhang et al. reported that rh-chemerin attenuates GMH-induced neuroinflammation by activating the CMKLR1/CAMKK2/AMPK pathway [22]. These results indicate that the Aβ/CMKLR1 axis may not affect the activation state of microglial cells. Moreover, a recent study found that, in a rat model of hypoxic-ischemic encephalopathy, rh-chemerin treatment reverses neurological impairments and alleviates neuronal apoptosis partially through the CMKLR1/CAMKK2/AMPK pathway [32]. Interestingly, oligomeric Aβ_42_ induces neuronal synaptic damage by activating the CAMKK2/AMPK/tau pathway [33]. These results indicate that the balance of the chemerin/CMKLR1/CAMKK2/AMPK and the Aβ/CAMKK2/AMPK pathways may play a pivotal role in maintaining neuronal and synaptic functions. These studies and our results suggest that enhancing the chemerin/CMKLR1 axis may prevent or alleviate the pathological process of AD.

Studies have reported that chemerin/CMKLR1 promotes chemotactic responses of many immune cells and non-immune cells, including macrophages, immature myeloid and plasmacytoid DCs, NK cells, vascular smooth muscle cells (SMCs), endothelial cells, and gastric cancer cells [13,34,35,36,37,38,39,40]. In the present study, we demonstrate for the first time that chemerin/CMKLR1 induced the migration and polarization of microglia by regulating actin and microtubule remodeling, and Golgi reorientation. Cell polarization is required for cellular movement and migration. It is characterized by changes in the cell morphology at the wound or chemoattractants, remodeling of actin filaments and microtubules, and reorientation of the Golgi apparatus to face the direction of migration [30,41]. Ramos-Junior et al. reported that chemerin/CMKLR1 induces the adhesion of osteoclasts to the bone surface by mediating F-actin reorganization [42]. The inhibition of CMKLR1 expression and F-actin polymerization suppresses the chemotaxis of plasmacytoid DCs toward chemerin [43]. In addition, further mechanism experiments confirmed that the p38 MAPK pathway participates in the promotion of chemerin/CMKLR1 on the migration and polarization of microglia. Kunimoto et al. reported that the chemerin/CMKLR1 axis induces vascular SMC migration and proliferation through the oxidant-dependent phosphorylation of Akt/ERK signals [44]. Chemerin/CMKLR1 is also involved in increased macrophage F-actin polymerization and localization to form the phagocytic cup in a Syk-dependent manner [45]. Another study found that rh-chemerin induces the formation of endothelial microtubules by activating MEK1, upstream from ERK1/2 MAPK [46]. All of these results indicate that the chemerin/CMLLR1 axis in different cell types exerts its biological functions through different downstream signal pathways.

In conclusion, the present study demonstrates that the chemerin/CMKLR1 axis plays a role in microglia migration. The chemerin/CMKLR1 axis promotes microglial migration and polarization by inducing the remodeling of actin filament and microtubulin, and Golgi reorientation. p38 MAPK pathway activation is involved in the promotion of the chemerin/CMKLR1 axis on microglial migration and polarization. Furthermore, the chemerin/CMKLR1 axis participates in the migration and recruitment of microglia to Aβ plaques/Aβ_42_ in vivo and in vitro. Modulation of the chemerin/CMKLR1 axis to balance overactivation of the Aβ/CMKLR1 axis is a new potential strategy for AD treatment.

## 4. Materials and Methods

### 4.1. Antibodies and Reagents

Dulbecco’s modified Eagle’s medium (DMEM), Iscove’s modified Dulbecco’s medium (IMDM), fetal bovine serum (FBS), and trypsin-ethylenediaminetetraacetic acid (trypsin-EDTA) were purchased from Gibco (Invitrogen, Carlsbad, CA, USA). Chemerin9 (C9), Chemerin15 (C15) peptides, and Aβ_42_ (≥95% purity) were synthesized at Shanghai Science Peptide Biologic Technology Co., Ltd., (Shanghai, China). Chemerin (recombinant human chemerin) was purchased from R&D systems (Minneapolis, MN, USA). Mouse anti-chemerin antibody was purchased from Abcam (Cambridge, UK). The BCA protein assay kit, 4,6-diamidino-2-phenylindole (DAPI), and FR180204 (ERK inhibitor) were obtained from the Beyotime Institute of Biotechnology (Nantong, China). Rabbit polyclonal anti-Iba1 was from FUJIFILM Wako Pure Chemical Corporation (Osaka, Japan). Rabbit polyclonal anti-α-tubulin and rabbit polyclonal anti-GM130 antibodies were from Sigma-Aldrich, Inc (St. Louis, MO, USA). Mouse monoclonal anti-CMKLR1, mouse polyclonal anti-CD11b, and mouse monoclonal anti-FITC-phalloidin antibodies were obtained from Santa Cruz Biotechnology (Dallas, TX, USA), BD Pharmingen (San Diego, CA, USA), and Merck KGaA (Darmstadt, Germany), respectively. AlexaFluor-488-conjugated anti-rabbit IgG secondary antibody and AlexaFluor-568-conjugated anti-mouse IgG secondary antibody were from Invitrogen. Other reagents were purchased from Sigma-Aldrich.

### 4.2. Animals

The APP/PS1 transgenic mice in C57BL/6J background (APP_SWE_/PS1ΔE9^+/−^, stock number 005864) were obtained from the Jackson Laboratory (Bar Harbor, ME, USA). The CMKLR1 knockout (*CMKLR1^−/−^*) mice in C57BL/6J background were generated by Shanghai Bioray Laboratory (Shanghai, China). The CMKLR1^−/−^ mice were crossed with the APP/PS1 mice to generate APP/PS1-*CMKLR1^+/−^* mice, and then, the latter was further crossed with the *CMKLR1^+/^*^−^ mice to create the following three groups: WT (APP/PS1^−/−^-*CMKLR1^+/+^*), APP/PS1 (APP/PS1^+/−^-*CMKLR1^+/+^*), and APP/PS1-*CMKLR1^−/−^* (APP/PS1^+/*−*^-*CMKLR1^−/−^*) [23]. Mouse genotypes were detected by PCR. All mice were housed (4–5 mice per cage) with a 12/12 h light/dark cycle, with ad libitum access to food and water. The housing, breeding, and animal experiments followed the National Institutes of Health Guide for the Care and Use of Laboratory Animals, with procedures approved by the Biological Research Ethics Committee, Shanghai Jiao Tong University. All three groups of male and female mice at the age of 9 months were sacrificed by decapitation, and their brains were removed immediately. The brain hemispheres were fixed with 4% paraformaldehyde (PFA) in 0.1M phosphate-buffered saline (PBS), followed by cryoprotection in 30% sucrose. Sagittal sections of 30 μm thickness were cut using a freezing sliding microtome. The sections were stored in glycol anti-freeze solution (12.5 g/L polyvinylpyrrolidone (average MW 40,000), 375 g/L saccharose, 375 mL/L glycol, and 625 mL/L Tris-buffered saline (TBS, 0.1 M, containing 12.1 g/L Tris-base, 40 g/L NaCl)) at −20 °C until immunofluorescence staining.

### 4.3. Microglial Cell Cultures

The primary microglial cultures were prepared from newborn (postnatal day 0) WT Sprague Dawley (SD) rat pups as previously described [47]. Briefly, cerebral cortices were removed from the brains of rats; then, the meanings and microvessels were removed. The tissues were minced with a sterile ophthalmic scissor and digested with 0.05% trypsin at 37 °C for 10 min. The cell suspension was filtered through a 40 μm sieve; then, the cells were plated on poly-D-lysine-coated 75 cm^2^ flasks with DMEM medium (containing 10% FBS, 100 U/mL penicillin, and 100 μg/mL streptomycin sulfate). The medium was replenished on day 1 and day 3. On day 7, microglial cells in the flasks were gently shaken off at 220 rpm for 3 h, and the medium containing detached microglia was collected. The collected cells were seeded in cell culture plates for future use. The purity of primary microglia was >95% as determined by immunocytochemistry with antibodies against CD11b [48].

The murine microglial cell line N9, originally from Dr. P. Ricciardi-Castagnoli (University of Milano-Bicocca, Italy), was kindly provided by Dr. Yingying Le (Institute for Nutritional Sciences, Shanghai Institutes for Biological Science, Shanghai, China). N9 cells were grown in IMDM supplemented with 10% FBS, 100 U/mL penicillin, and 100 μg/mL streptomycin sulfate.

### 4.4. Western Blot

The mouse brain tissues were homogenized in lysis buffer including 50 mM Tris-HCL (pH 7.4), 100 mM NaF, 2 mM EDTA, 10 mM β-mercaptoethanol, 2 mM NaVanadate, 8.5% sucrose, 5 μg/mL aprotinin, 100 μg/mL leupeptin, and 5 μg/mL pepstatin. The protein concentrations were detected using BCA Kits according to the manufacturer’s protocol. The quantitative homogenates were added to a 5× sodium dodecyl sulfate (SDS)-PAGE loading buffer, heated for 10 min at 99 °C, and separated onto 10% or 12% SDS-PAGE. After separation, the samples were transferred onto nitrocellulose membranes (GE Healthcare, Wauwatosa, WI, USA), and the membranes were blocked with 5% non-fat milk for 1 h at room temperature. Then, the membranes were incubated with primary antibodies including anti-Iba1 (1:1000), anti-chemerin (1:1000), anti-CMKLR1 (1:1000), and anti-GAPDH (1:20,000), followed by IRDye^®^ 800CW or IMDye^®^ 800CW secondary antibodies. The membranes were scanned using an Odyssey P140-CLx Infrared Imaging System (LI-COR, Inc., Lincoln, NE, USA). Densitometric quantification of protein bands was analyzed by the ImageJ software (National Institutes of Health, Bethesda, MD, USA).

### 4.5. Immunofluorescence Staining

Sections of the mouse brain were processed for standard immunofluorescence staining [21]. Briefly, the sections were washed with 0.05 M TBS, permeabilized with 0.1% Triton X-100 in TBS for 10 min, and blocked with 5% normal goat serum in TBS (0.1% Tween-20) for 30 min at room temperature. The sections were incubated overnight at 4 °C with anti-Iba1 antibody (1:500) or anti-chemerin (1:200) in TBS. After rinsing with TBS, the samples were incubated with Alexa Fluor 488-conjugated anti-rabbit secondary antibody (1:500) or Alexa Flour 568-conjugated anti mouse secondary antibody (1:500) at room temperature for 1 h. For double immunofluorescence staining of Iba1 and thioflavin-S (Thio-S), the staining of Iba1 was performed as described above. The sections were rinsed in TBS and stained with 1% Thio-S for 10 min at room temperature. Then, the sections were washed with 50% ethanol twice. After washing three times in TBS, the sections were stained for nuclei with 100 ng/mL of DAPI and mounted on glass slides.

The fluorescent confocal images were captured using a laser-scanning confocal fluorescent microscope (TCS SP8, Leica Micro-systems, Wetzlar, Germany). The relative immunofluorescence intensity of Iba1 was quantified using the ImagePro Plus Software (Media Cybernetics, Silver Spring, MD, USA). The number of microglia (Iba1 staining, green fluorescence) colocalized with Aβ plaques (red fluorescence) was counted. The data are presented as the means ± SEM based on four individual fields for each region, using 3–4 mice in each group, or from at least three independent experiments, each in triplicate.

### 4.6. Boyden Chamber Assay

The Boyden chamber assay was performed as described previously [27]. Standard 48-well chemotaxis chambers (Neuro Probe, Gaithersburg, MD, USA), in which the upper and lower wells were separated by a polycarbonate membrane (8 μm pore size), were used to study the migration of microglial cells. The lower wells of the chamber were added to either control media or media containing various concentrations of chemerin, C9, or 10% FBS. The membrane was an 8 μm pore size polycarbonate filter (Neuro Probe) over the lower wells of the chamber. The upper chamber wells were filled with primary microglia or N9 cells with or without 15 min pretreatments with C15 (1 μM), SB203580 (10 μM), FR180204 (5 μM), SP600125 (5 μM), or LY294002 (5 μM). The chamber was incubated in a humidified incubator with 5% CO_2_ at 37 °C for 12 h; then, the filter was carefully removed. Microglia on the upper surface of the filter that did not migrate were wiped off with a cotton-tipped swab. The remaining cells that migrated to the bottom surface of the filter were fixed with methanol, stained with 0.1% crystal violet, and counted with a phase contrast inverted microscope (IX51, Olympus Optical Co. Ltd., Tokyo, Japan) custom-fitted with a digital camera (EOS 1100D, Canon Inc., Tokyo, Japan). The results were expressed as the chemotaxis index to represent the fold increase or decrease in the number of migrated cells in response to the chemoattractant over that of the control medium. The data are presented as the means ± SEM from three independent experiments, each with three wells for each group.

### 4.7. Scratch-Wound Assay

The scratch-wound assay is a method commonly used to assess and quantify the migrating capacity of cells. The rationale for the wound healing assay is to disrupt the confluent cell monolayer, creating a cell-free region to simulate a wound, called a “scratch”, which is available for cellular migration and repair. During wound healing, cells at the wound edge polarize and migrate to the wound space [49]. The scratch-wound assay was performed as described previously [50]. A scratch was made in confluent primary cultures of microglia or N9 cells grown on poly-D-lysine-precoated coverslips using a 10 μL pipette tip; then, a 500 μm-wide cell-free area was generated. The movement images of the cells were taken under light microscopy (Olympus Optical Co. Ltd., Tokyo, Japan). Images were captured at 0 h as the control. The cells were incubated with chemerin (5 nM) or C9 (100 nM) with or without 15 min pretreatments with SB203580 (10 μM), FR180204 (5 μM), SP600125 (5 μM), or LY294002 (5 μM) for 8 h, 12 h, or 16 h. Then, the pictures were captured at each of the time points. In each image, the distance between one side of the scratch and the other was examined using ImageJ. The distance of each scratch closure was obtained by comparing the images from time 0 to each time point. The migration distance at the same time interval indicated the migrating capacity of cells. The results were quantified by calculating the mean migrated distance of leading cells in the scratched area.

Protrusion formation was examined at 16 h after scratching following fixation and F-actin and microtubule staining [51,52]. In order to detect actin and microtubule networks, the immunofluorescence staining was conducted as described above after scratch-wound assay. The cells were stained with anti-FITC-phalloidin (1:1000) for F-actin and anti-α-tubulin (1:1000) for microtubule at 16 h after scratching. The secondary antibody Alexa Fluor 488-conjugated anti-rabbit antibody (1:500) against the anti-α-tubulin was used. The primary cultures of microglia with staining were scored as protruding when their protrusions were at least 4 times longer than wide.

To analyze the Golgi reorientation, the cells were fixed and stained with anti-GM130 antibody (1:2000) at 12 h after scratching. The secondary antibody Alexa Fluor 488-conjugated anti-rabbit antibody (1:500) against the anti-GM130 antibody was used. The immunofluorescence staining was performed as described above. The location of Golgi in front of the nucleus, within 120° sectors facing the wound, was considered positive.

### 4.8. Measurement of Microglial Clusters

Microglial N9 cells (1 × 10^4^) were incubated with 3 μM fibrillar Aβ_42_ with or without 5 nM chemerin or 100 nM C9 in a 24-well plate for 24 h. The incubation of p38 (SB203680) was performed 15 min before Aβ_42_ treatment with chemerin (5 nM) or C9 (100 nM). The distribution of aggregated microglial N9 cells was captured with a phase contrast inverted microscope custom-fitted with a digital camera. The cluster of microglia was defined as at least 10 cells aggregated together around Aβ_42_ [53]. The data are presented as the means ± SEM from three independent experiments, each with three wells for each group.

### 4.9. Statistical Analyses

All data are presented as means ± SEM. Two-group comparisons were evaluated by the two-tailed *t* test. Multiple comparisons were analyzed using one-way ANOVA, followed by Tukey’s post hoc test. All analyses were performed with the statistical software GraphPad Prism 8 (San Diego, CA, USA). *p* < 0.05 was considered statistically significant.

## Data Availability

All datasets generated or analyzed during this study are available from the corresponding author upon reasonable request.

## Supplementary Materials

The following supporting information can be downloaded at https://www.mdpi.com/1422-0067/23/16/9041/s1.
