# Correction: Chen et al. The Chemerin/CMKLR1 Axis Is Involved in the Recruitment of Microglia to Aβ Deposition through p38 MAPK Pathway. *Int. J. Mol. Sci.* 2022, *23*, 9041

**DOI:** 10.3390/ijms24010506

**Published:** 2022-12-28

**Authors:** Yanqing Chen, Zhen Liu, Ping Gong, Haibo Zhang, Yijun Chen, Songquan Yao, Wei Li, Yan Zhang, Yang Yu

**Affiliations:** School of Pharmacy, Shanghai Jiao Tong University, Shanghai 200240, China

In the original publication [1], there was a mistake in Figure 3H as published. The representative picture of the chemerin + C15 treatment group in Boyden chamber assay was wrong, and this error was due to an unintentional mistake in the selection of representative images. These changes do not affect the conclusion and findings since the quantitative statistics were obtained based on the correct raw data. The corrected Figure 3 appears below.

The authors apologize for any inconvenience caused and state that the scientific conclusions are unaffected. This correction was approved by the Academic Editor. The original publication has also been updated.

## Figures and Tables

**Figure 3:**
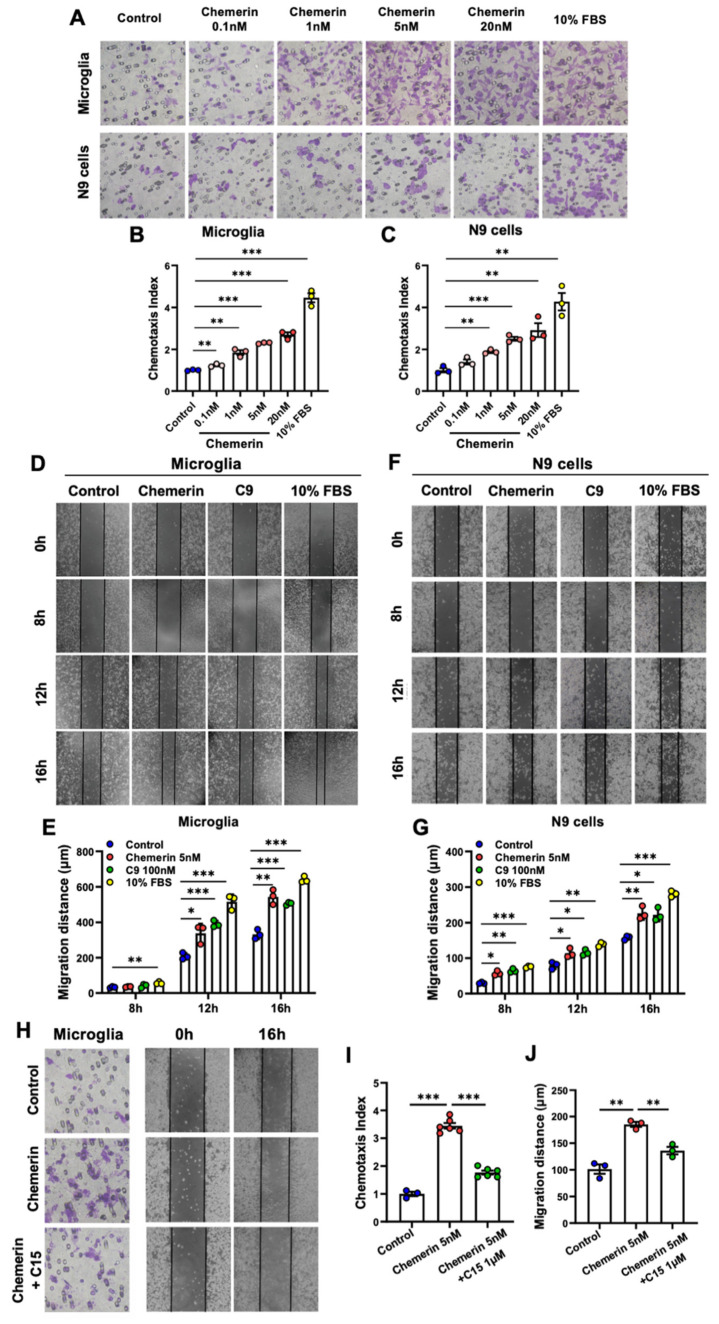
Chemerin/CMKLR1 axis promotes the migration of microglia in Boyden chamber migration and scratch-wound assay. (**A**) Primary cultures of microglia and murine microglial N9 cells were incubated for 12 h with chemerin (0.1–20 nM) or 10% FBS, and the migration of the cells was evaluated by 48-well chemotaxis chambers. Representative images of migrated cells on membrane filters are shown. (**B**,**C**) The Quantified data are shown. Magnification, ×400. Primary microglia and N9 cells were treated with chemerin (5 nM), C9 (100 nM), or 10% FBS; then, the migration of the cells was detected by scratch-wound assay. The microglia were photographed at 0 h, 8 h, 12 h, and 16 h. Representative images of migrated primary microglia and N9 cells are shown in (**D**,**F**), and quantified data are shown in (**E**,**G**), respectively. Magnification, ×100. (**H**) Primary microglia were incubated with chemerin (5 nM) with or without a 15 min pretreatment with C15 (1 μM). After 16 h incubation, the migration of microglia was detected by 48-well chemotaxis chambers and scratch-wound assay. Magnification, ×400 and ×100, respectively. The quantified data were shown in (**I**,**J**). The results are expressed as the mean ± SEM from three separate experiments, each in at least triplicate. * *p* < 0.05, ** *p* < 0.01, *** *p* < 0.001.

## References

[1] Chen Y., Liu Z., Gong P., Zhang H., Chen Y., Yao S., Li W., Zhang Y., Yu Y. (2022). The Chemerin/CMKLR1 Axis Is Involved in the Recruitment of Microglia to Aβ Deposition through p38 MAPK Pathway. Int. J. Mol. Sci..

