# Supplementary material for: The Chemerin/CMKLR1 Axis Is Involved in the Recruitment of Microglia to Aβ Deposition through p38 MAPK Pathway
Source: Int J Mol Sci. 2022 Aug 12;23(16):9041. doi: 10.3390/ijms23169041 (PMC9409288; doi:10.3390/ijms23169041)
Supplement: Supplementary file 1 [file ijms-23-09041-s001.zip › Supplementary file.pdf]

SUPPLEMENTARY MATERIALS

Supplementary Methods:

Cell viability assay

Microglial cell viability was detected by a colorimetric assay with methyl thiazolyl tetrazolium (MTT). Primary cultures of microglia and microglial N9 cells grown in 96-well plates were exposed to chemerin (20 nM), C9 (100 nM), SB203580 (5 μM or 10 μM), FR180204 (5 μM or 10 μM), SP600125 (5 μM or 10 μM), or LY294002 (5 μM or 10 μM) for 12 h or 16 h. The culture medium was removed and the cells were incubated with MTT (1 mg/ml) at 37 °C for 4 h. After three washes with phosphate-buffered saline (PBS, pH 7.4), the insoluble formazan product was dissolved in dimethyl sulfoxide. The optical density at 570 nm was determined on a FlexStation III plate reader (Molecular Devices, Mountain View, CA). Cell viability was expressed as a percentage of control.

Supplementary Figures

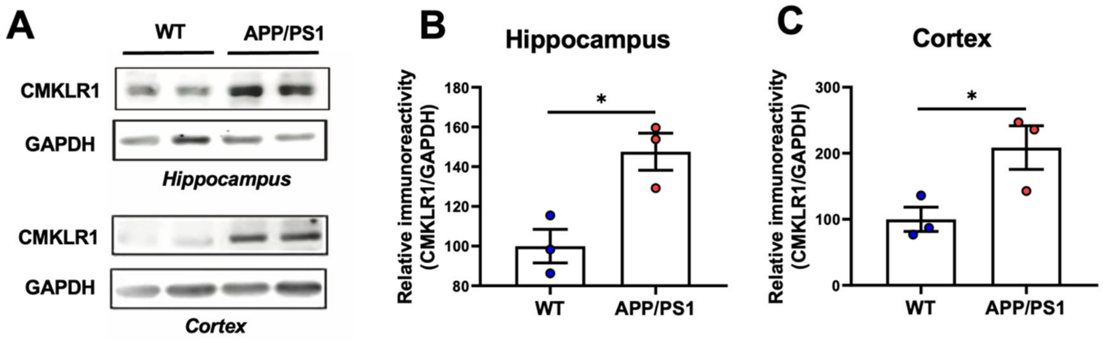

Supplementary Figure S1. Increase of CMKLR1 in the hippocampus and cortex of APP/PS1 transgenic mice. (**A**) Representative Western blot showing the expression of CMKLR1 in the hippocampus and cortex in 9-month-age WT and APP/PS1 mice. (**B, C**) Quantification of immunoreactivity of the blots, normalized against GAPDH. The results are expressed as the mean ± SEM using at least 3 mice in each group. $*p < 0.05$

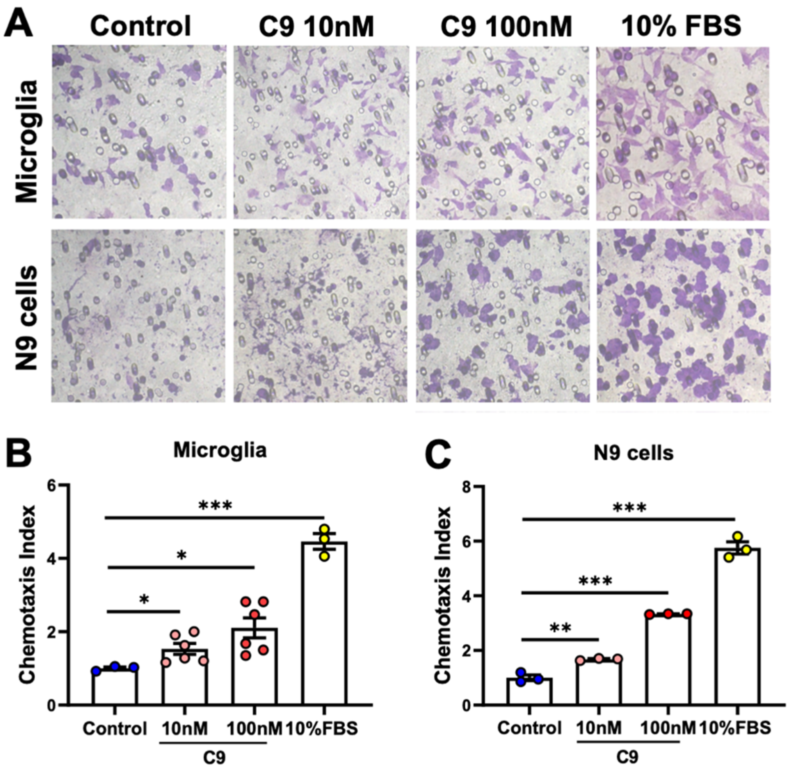

Supplementary Figure S2. C9 promotes the migration of primary microglia and murine microglial N9 cells in Boyden chamber migration assay. Primary microglia and N9 cells were incubated for 12 h with C9 (10 nM or 100 nM) or 10% FBS, and the migration of microglial cells was evaluated by 48-well chemotaxis chambers. Representative images of migrated cells on membrane filters were shown in (**A**) and quantified data were shown in (**B, C**). The results are expressed as the mean ± SEM from three separate experiments, each in at least triplicate. Magnification, × 400. *$p < 0.05$, **$p < 0.01$, ***$p < 0.001$

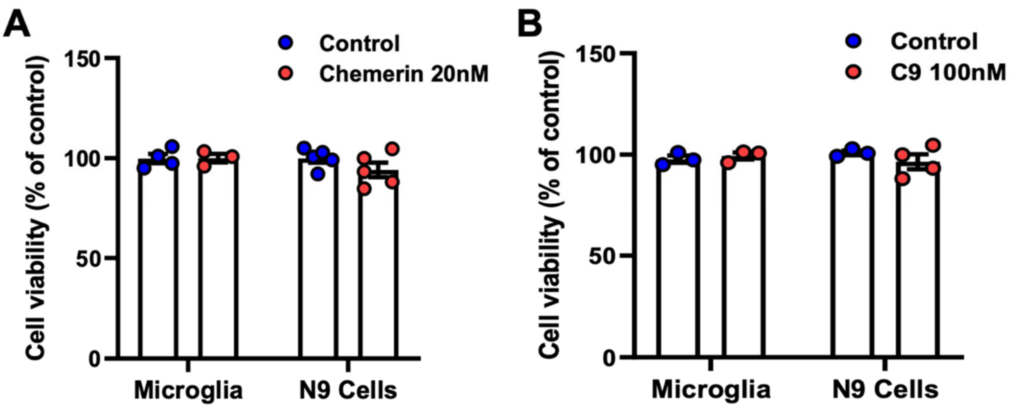

Supplementary Figure S3. Effect of chemerin and C9 on cell viability of primary microglia and N9 cells. (**A, B**) Primary cultures of microglia and N9 cells were exposed to chemerin (20 nM) or C9 (100 nM). Microglial cell viability was detected after 16 h by MTT. Data are expressed as percentage of surviving cells over control cells. Results are expressed as the mean ± SEM based on three independent experiments, each in at least triplicate.

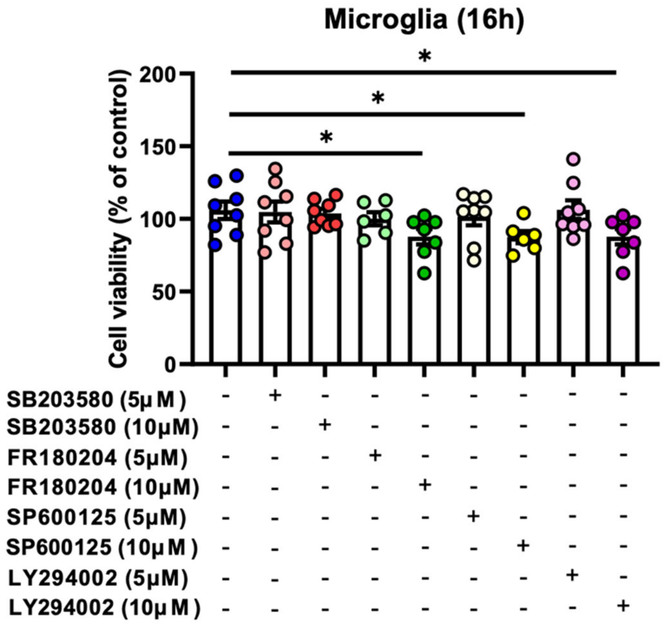

Supplementary Figure S4. Effect of MAPKs and PI3K inhibitors on cell viability of primary microglia. Primary cultures of microglia were exposed to SB203580 (5 or 10 μM), FR180204 (5 or 10 μM), SP600125 (5 or 10 μM), or LY294002 (5 or 10 μM), and the cell viability was detected after 16 h by MTT. Data are expressed as percentage of surviving cells over control cells. Results are expressed as the mean ± SEM based on three independent experiments, each in at least triplicate.

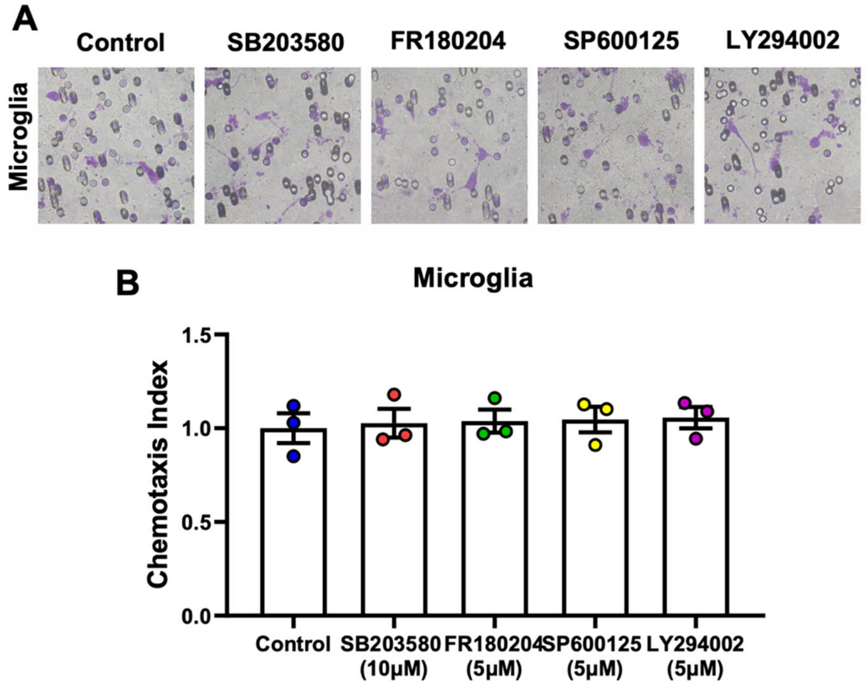

Supplementary Figure S5. Effect of MAPKs and PI3K inhibitors on the migration of primary microglia. Primary cultures of microglia were incubated for 12 h with SB203580 (10 μM), FR180204 (5 μM), SP600125 (5 μM), or LY294002 (5 μM). The cell migration was determined by 48-well chemotaxis chambers. Representative images of migrated cells on membrane filters were shown in (**A**) and quantified data were shown in (**B**). Magnification, × 400. Results are expressed as the mean ± SEM based on three independent experiments, each in at least triplicate.

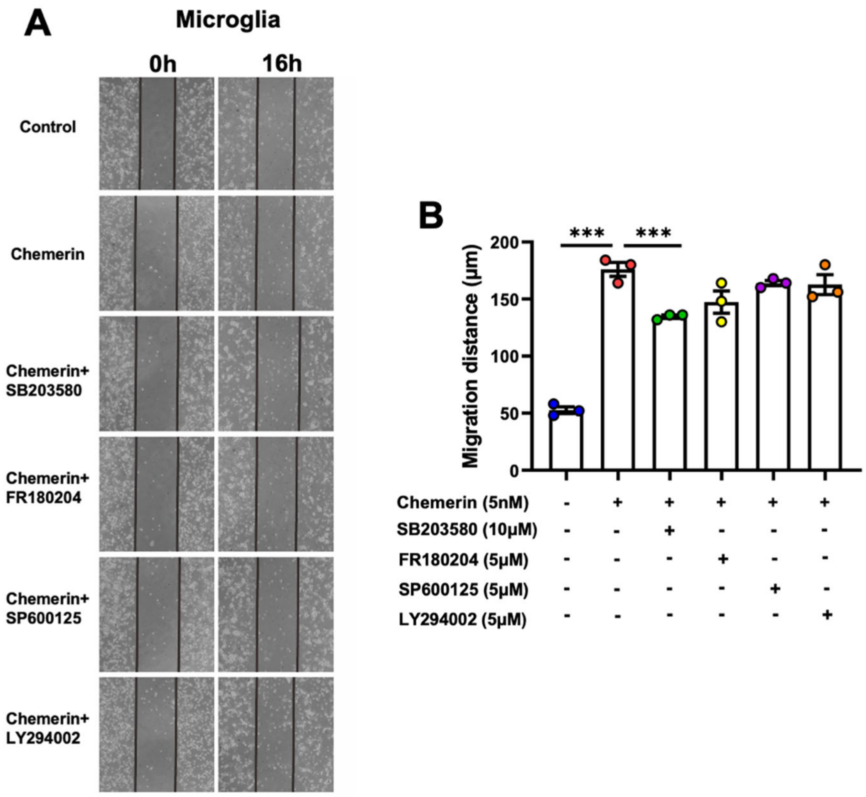

Supplementary Figure S6. Inhibiting p38 suppresses the promotion of chemerin on the migration of primary microglia in scratch-wound assay. Primary cultures of microglia were incubated with chemerin (5 nM) with or without a 15 min pretreatment with SB203580 (10 μM), FR180204 (5 μM), SP600125 (5 μM), and LY294002 (5 μM), and then the migration of the cells was detected by scratch-wound assay. Microglia were photographed at 0 h and 16 h. Representative images of migrated primary microglia were shown in (**A**), and quantified data were shown in (**B**). The results are expressed as the mean ± SEM from three separate experiments, each in at least triplicate. Magnification, × 100. ***$p < 0.001$
